# Eventration of Diaphragm Presenting as Small Bowel Obstruction

**Published:** 2010-12-01

**Authors:** Muhammad Ahmed, Bilal Mirza, Afzal Sheikh

**Affiliations:** Department of Pediatric Surgery, The Children's Hospital and the Institute of Child Health Lahore, Pakistan

**Dear Sir**

Eventration of diaphragm is a well known entity that may present at any age. Most of the cases are diagnosed in first decade of life. The usual clinical presentation is that of respiratory distress and recurrent respiratory tract infections; however unusual presentations have also been reported [1]. The objective of this report is to highlight a rare possibility of small bowel obstruction in settings of ineffective or incomplete separation of abdominal and thoracic cavities by diaphragm. A case of left sided eventration of diaphragm presenting with small bowel obstruction is being reported.

A 6-month-old infant presented to our institution with complaints of abdominal distension, bilious vomiting, non passage of stool, and irritability for two days. The past medical history was insignificant. On general physical examination the patient was vitally stable. Abdominal examination revealed mildly distended and tender abdomen in all quadrants with absent bowel sounds. A digital rectal examination revealed only mucous in the rectum. Laboratory investigations were within normal limits. Abdominal radiographs, in supine and erect postures, delineated intestinal air shadows moving up into the left hemithorax and air fluid levels respectively (Fig. 1,2). An exploratory laparotomy was performed that revealed few loops of small and large bowel adherent with each other and also with the eventration, causing small bowel obstruction at the level of distal ileum. Adhesiolysis relieved the obstruction following which plication of the diaphragm was performed. Ladd’s procedure was added for the associated malrotation. Patient had an uneventful postoperative recovery. He has a follow up of 6 months and doing well.

**Figure:**
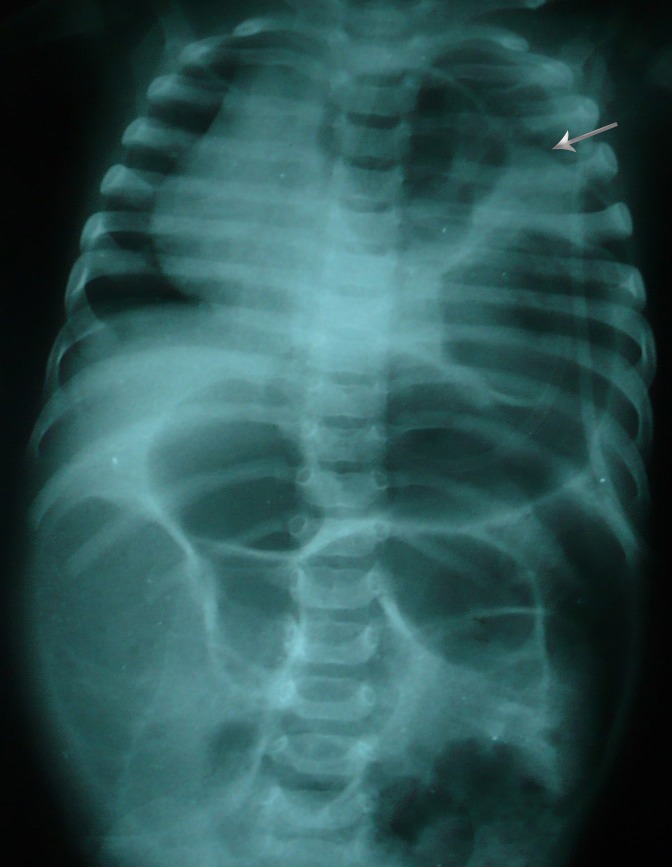
Figure 1: Supine radiograph showing dilated bowel loops entering into the left hemithorax. An eventration of diaphragm can also be appreciated (Arrow).

**Figure:**
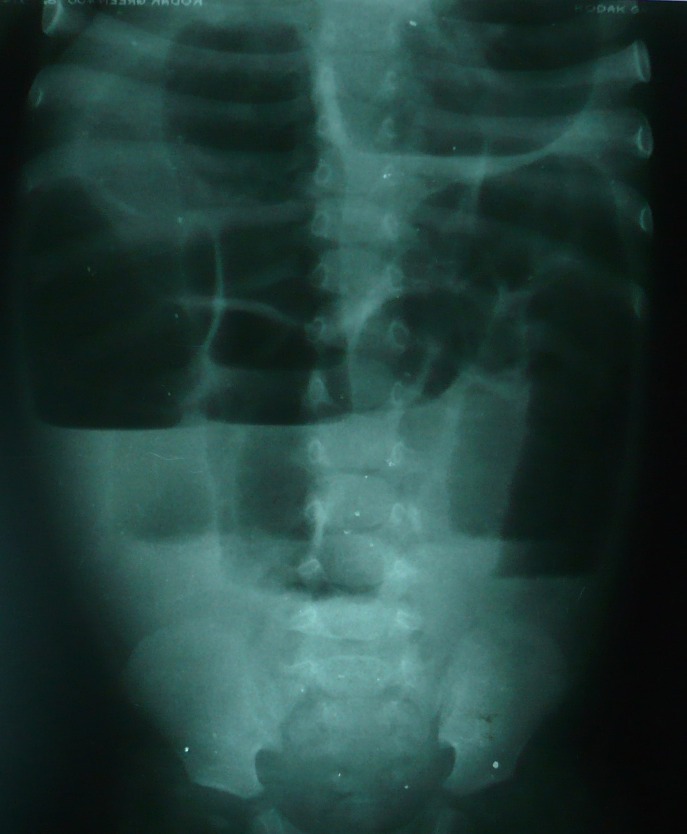
Figure 2: Erect radiograph showing multiple air fluid levels.

Eventration of diaphragm is an abnormal elevation of the dome of diaphragm; usually unilateral and left sided, but isolated right sided and bilateral involvements have also been reported. The lesion may be congenital or acquired as to the etiology. Embryologically, a failure of myoblastic migration to diaphragm or of its innervation, during 7th week of gestation, results in a thin and hypoplastic diaphragm on the involved side [2].

During the same period of development the physiologically herniated bowel start returning to the abdominal cavity. The eventration of diaphragm impairs the process of normal rotation and fixation as happens in case of congenital diaphragmatic hernia; thus resulting in association of malrotation as found in our case. Acquired eventration always develops due to phrenic nerve palsy in conditions such as obstructed labor, surgery (iatrogenic) etc [2,3].

The treatment can be delayed in asymptomatic patients. At times patients with eventration of diaphragm may present with life threatening complications necessitating urgent surgical intervention. These conditions are acute gastric volvulus, severe respiratory distress, and intestinal obstruction. The management includes plication of the eventration and the management of the complications, as done in case reported here [3,4]. 

It is assumed that the associated malrotation and malfixation of intestine caused recurrent episodes of partial intestinal volvulus and ischemia, resulting in inflammation and adhesions between intestinal loops and the diaphragm.

Few cases of intestinal obstruction after spontaneous rupture of congenital eventration of diaphragm have been reported in literature; three cases of intestinal obstruction after plication of diaphragm have also been documented [5,6].

Small bowel obstruction as a sole presentation of eventration of diaphragm in pediatric patients is a very rare event. The treating physician and surgeon must keep this possibility in mind.

## Footnotes

**Source of Support:** Nil

**Conflict of Interest:** None declared
